# Venous Septal Alcohol Ablation as a Salvage Strategy in Obstructive Hypertrophic Cardiomyopathy

**DOI:** 10.1016/j.jaccas.2026.108609

**Published:** 2026-06-03

**Authors:** Oguz Ucar, Ozcan Ozdemir

**Affiliations:** Department of Cardiology, Etlik City Hospital, Ankara, Turkey

**Keywords:** alcohol septal ablation, case series, hypertrophic cardiomyopathy, septal reduction therapy, venous approach

## Abstract

Septal alcohol ablation (SAA) is an established septal reduction therapy for symptomatic obstructive hypertrophic cardiomyopathy (HCM); however, its efficacy depends on favorable septal arterial anatomy. In a subset of patients, arterial access may be limited by unfavorable anatomy or prior septal artery occlusion, rendering conventional SAA ineffective. We report the first case series describing venous SAA as an alternative and salvage strategy. Two symptomatic men with obstructive HCM underwent venous septal ethanol delivery after inadequate arterial septal ablation. In the first case, limited gradient reduction after arterial SAA prompted adjunctive venous ethanol infusion via the coronary sinus, resulting in sustained hemodynamic improvement. In the second case, prior arterial SAA had led to septal artery occlusion; repeat arterial intervention failed, and venous septal ethanol infusion using a controlled balloon-isolated technique achieved immediate and durable gradient reduction. At the 6-month follow-up, both patients demonstrated marked symptomatic improvement and persistent left ventricular outflow tract gradient reduction without conduction disturbances. Venous SAA may represent a feasible salvage strategy in carefully selected patients with obstructive HCM.

## Visual Summary

**Figure:**
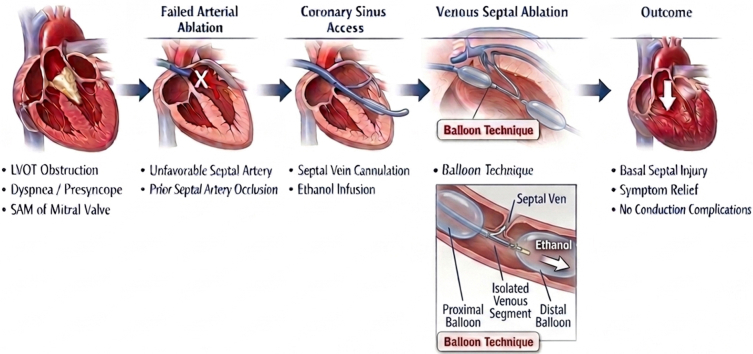

Obstructive hypertrophic cardiomyopathy (HCM) is characterized by dynamic left ventricular outflow tract (LVOT) obstruction caused by asymmetric septal hypertrophy and systolic anterior motion (SAM) of the mitral valve. In patients with persistent symptoms despite optimal medical therapy, septal reduction therapy, either surgical myectomy or septal alcohol ablation (SAA), is recommended in experienced centers.^1^^,^^2^ SAA was first introduced as a catheter-based alternative to surgical septal myectomy and has since become an established therapy in selected patients with obstructive HCM.^3^^,^^4^

In routine clinical practice, suitable septal arterial anatomy may be absent because of a small vessel caliber, unfavorable perfusion territory, or prior septal artery occlusion after previous SAA. In such circumstances, effective arterial ethanol delivery may not be achievable.

Venous ethanol delivery through the coronary venous system has been described in electrophysiology practice for treatment of ventricular tachycardia originating from intramural myocardial substrates as well as for vein-of-Marshall ethanol infusion during atrial fibrillation ablation. These observations support the feasibility of controlled ethanol infusion through the coronary venous circulation.^5^, ^6^, ^7^ We present a novel approach using venous SAA via the coronary venous system as a potential alternative and salvage strategy in patients with obstructive HCM when conventional arterial approaches are ineffective.

## Case Series

### Case 1

A 38-year-old man presented with exertional dyspnea and presyncope despite maximally tolerated beta-blocker therapy (metoprolol: 300 mg/d), corresponding to NYHA functional class III. Transthoracic echocardiography demonstrated asymmetrical septal hypertrophy with a septal thickness of 19 mm and SAM of the mitral valve, resulting in a resting LVOT gradient of 55 mm Hg. Cardiac magnetic resonance imaging showed no late gadolinium enhancement, with septal thickness measured at 20 mm at the midventricular level and persistent SAM.

**Figure 1:**
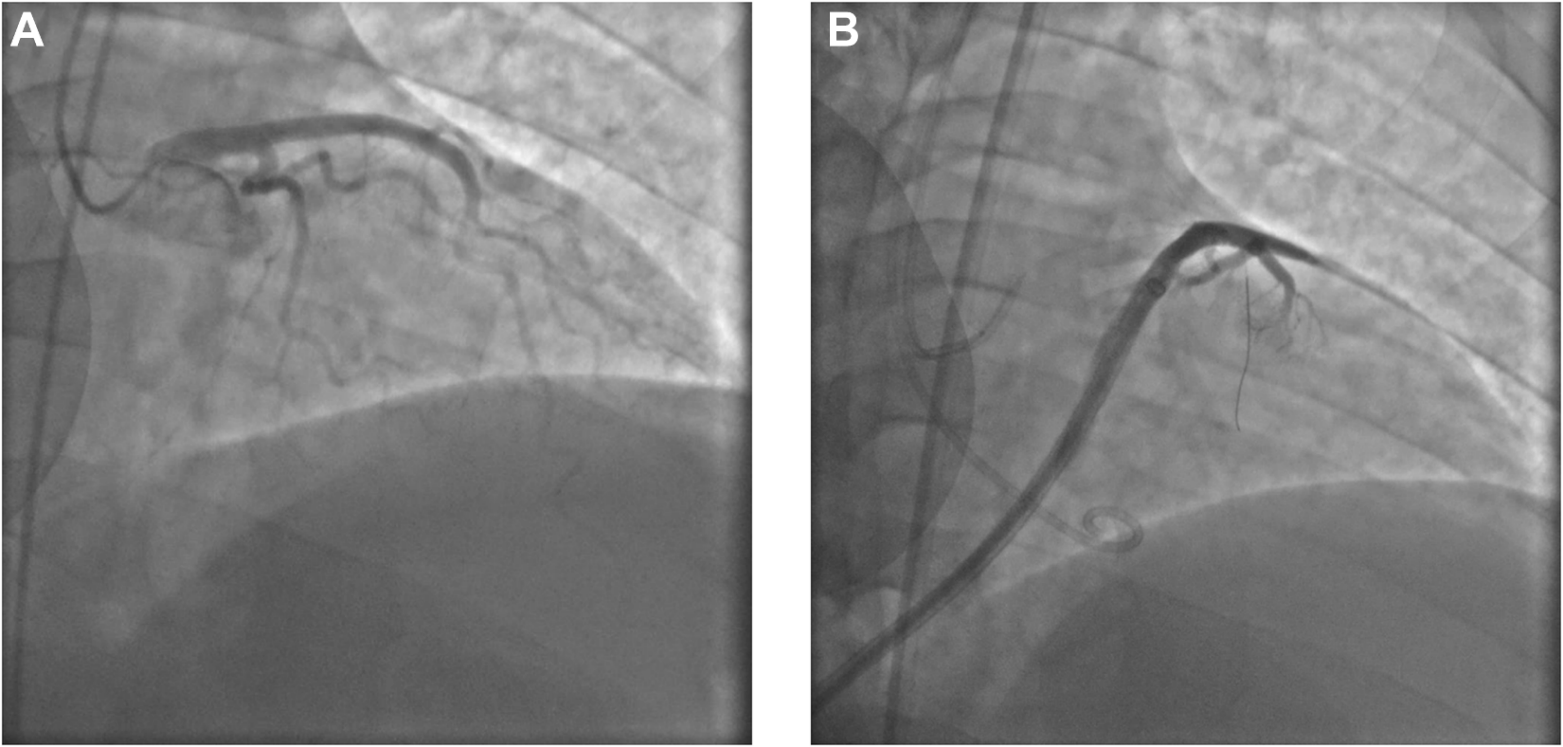
Baseline Coronary Angiography and Venous Cannulation (A) Coronary angiography demonstrated 2 small septal perforator arteries supplying the basal septum, both of limited caliber in case 1. (B) A microcatheter was navigated into the target septal vein, and 5 mL of ethanol was infused in case 1.

Invasive hemodynamic assessment before intervention revealed a resting LVOT gradient of 120 mm Hg. Coronary angiography demonstrated 2 small septal perforator arteries supplying the basal septum, both of limited caliber (Figure 1A). The second septal artery was crossed with a guidewire, and a 1.5-mm over-the-wire balloon was positioned proximally for occlusion. A total of 1.5 mL of ethanol was slowly injected; however, only partial reduction of the LVOT gradient was achieved, and no additional suitable septal arteries were identified.

Surgical septal myectomy was discussed with the patient as an alternative septal reduction strategy. However, after shared decision-making, the patient declined surgery and preferred a catheter-based treatment approach.

Because of insufficient hemodynamic response and absence of alternative arterial targets, a venous strategy was pursued. A coronary sinus catheter was advanced, and venography identified a septal vein draining the basal septum. A microcatheter was navigated into the target septal vein, and 5 mL of ethanol was infused in small aliquots over several minutes under continuous hemodynamic monitoring (Figure 1B). No arrhythmias or conduction disturbances occurred during or after the procedure.

After venous ethanol infusion, the resting LVOT gradient decreased to 35 to 40 mm Hg. The patient remained clinically stable without procedural complications. At the 6-month follow-up, the patient reported marked symptomatic improvement, with no recurrence of presyncope, and transthoracic echocardiography demonstrated a resting LVOT gradient of 32 mm Hg while the same medical therapy was continued.

### Case 2

A 51-year-old man presented with presyncope and exertional chest pain corresponding to NYHA functional class II. Transthoracic echocardiography demonstrated asymmetrical septal hypertrophy, with septal and posterior wall thicknesses of 18 mm and 16 mm, respectively. SAM of the mitral valve was present. The resting LVOT gradient was 95 mm Hg and increased to 136 mm Hg during the Valsalva maneuver. The patient was being treated with metoprolol 50 mg twice daily.

**Figure 2:**
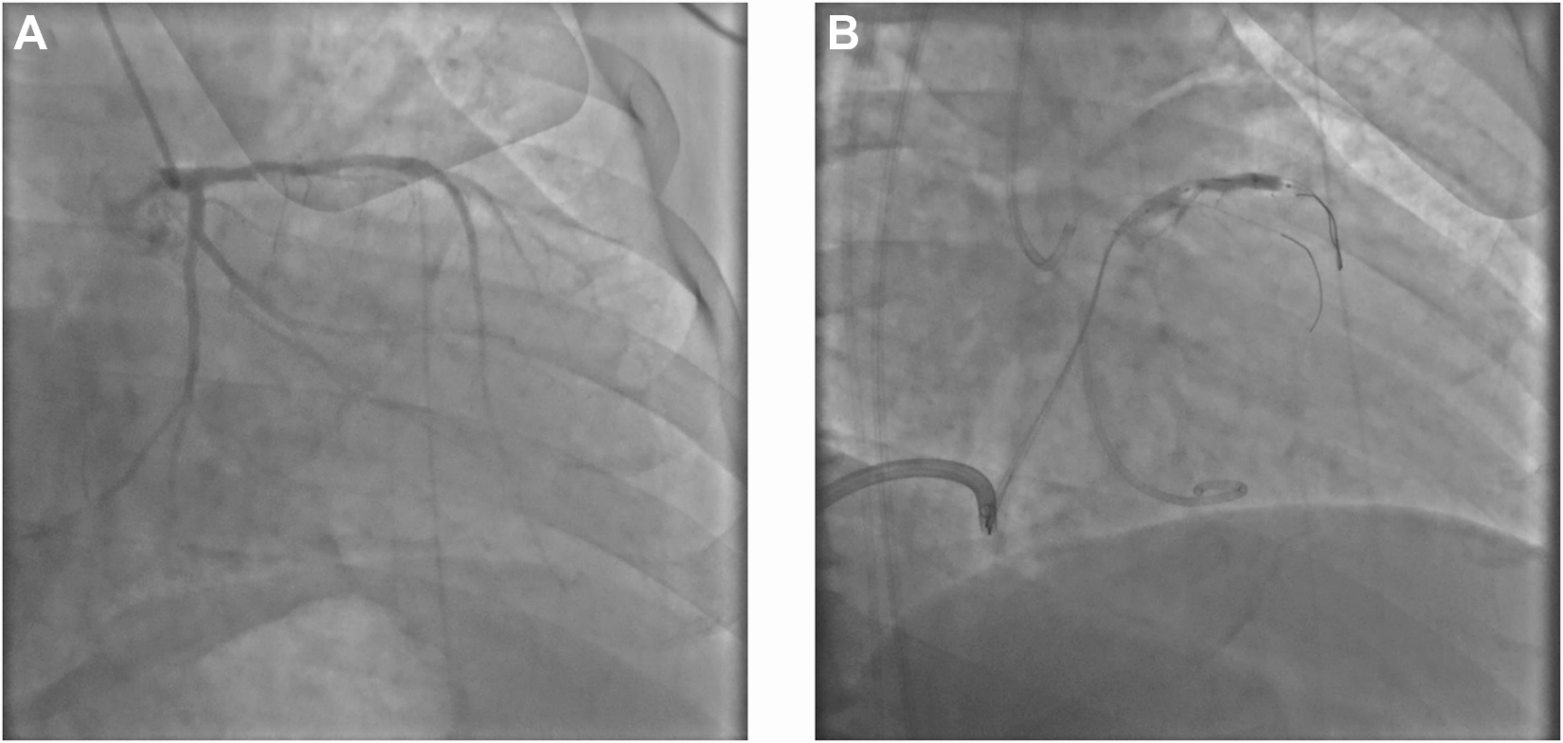
Coronary Angiography and Balloon-Isolated Venous Ablation (A) Coronary angiography revealed occlusion of the first septal perforator artery and thinner second septal artery in case 2. (B) A Caravel microcatheter was positioned between the 2 inflated balloons, creating an isolated venous segment. With both balloons inflated to prevent reflux, 2 mL of ethanol was infused in case 2.

The patient had a history of SAA performed in 2022. Coronary angiography revealed occlusion of the first septal perforator artery. Although a thinner second septal artery was cannulated and ethanol was injected, simultaneous invasive pressure measurements and echocardiographic assessment demonstrated no meaningful reduction in the LVOT gradient (Figure 2A).

In light of failed arterial reintervention, a venous approach was selected. Using femoral venous access, an Agilis steerable catheter (Abbott) was advanced into the coronary sinus, and coronary sinus venography was performed. Because of an unfavorable angle, direct cannulation of the septal vein was not feasible. A 3.0 × 10-mm balloon was advanced distally within the coronary sinus, followed by placement of a 3.5 × 10-mm balloon proximally. A Caravel microcatheter (Asahi Intecc) was positioned between the 2 inflated balloons, creating an isolated venous segment. With both balloons inflated to prevent reflux, 2 mL of ethanol was infused on 3 occasions (total volume: 6 mL) in a controlled fashion (Figure 2B).

A postprocedural invasive assessment demonstrated elimination of the resting LVOT gradient, with a residual gradient of 20 to 30 mm Hg during the Valsalva maneuver. The procedure was completed without complications. At the 6-month follow-up, transthoracic echocardiography demonstrated a resting LVOT gradient of 20 mm Hg, increasing to 40 mm Hg with Valsalva. No arrhythmic or conduction-related complications were observed.

## Rationale for Venous SAA

These 2 cases highlight complementary indications for venous septal ethanol delivery. In the first case, venous ablation served as an adjunctive strategy after suboptimal arterial SAA. In the second case, prior arterial SAA resulted in septal artery occlusion, rendering repeat arterial intervention ineffective. In both scenarios, the coronary venous system provided an alternative pathway to access the basal septum, enabling targeted myocardial injury when arterial supply to the relevant septal region was inadequate.

## Discussion

This case series describes venous SAA as a novel salvage strategy for septal reduction in obstructive HCM. Although arterial SAA remains an established catheter-based alternative to surgical myectomy, its success depends on the presence of suitable septal perforator arteries supplying the basal septum.^1^^,^^2^^,^^8^ In clinical practice, unfavorable septal anatomy, small vessel caliber, or prior septal artery occlusion may limit the feasibility of repeat arterial ablation.^9^

Venous ethanol delivery offers a conceptual advantage by approaching the target myocardium from the drainage side of the coronary circulation. Similar approaches have been reported in electrophysiology practice, where ethanol infusion through the coronary venous system has been used to treat ventricular tachycardia arising from intramural myocardial substrates and to facilitate vein-of-Marshall ablation during atrial fibrillation procedures.^5^, ^6^, ^7^ These experiences support the feasibility of controlled ethanol infusion via the coronary venous circulation.

Potential complications of septal ethanol ablation, including atrioventricular conduction disturbances requiring permanent pacemaker implantation, ventricular septal defect formation, and transient or persistent left ventricular dysfunction, were discussed with both patients before the procedure. These risks are well recognized in conventional arterial SAA and were considered during procedural planning.^3^^,^^4^^,^^10^

Surgical septal myectomy was discussed as an alternative septal reduction therapy; however, the patients declined surgical intervention and preferred a catheter-based strategy. Current clinical guidelines recommend septal reduction therapy, including surgical myectomy or SAA, for patients with obstructive HCM who remain symptomatic despite optimal medical therapy.^1^^,^^2^ The management of symptoms in HCM has evolved over time, with septal reduction therapies playing a central role in patients with drug-refractory obstruction.^11^ In addition, pharmacological options such as cardiac myosin inhibitors (eg, mavacamten) have recently emerged as potential therapies for symptomatic obstructive HCM. However, this therapy was not available in our country at the time the procedures were performed.^1^^,^^2^

The feasibility of venous septal ethanol delivery may depend on septal venous anatomy. Large venous channels or extensive collateralization could theoretically increase the risk of nontarget ethanol distribution, whereas smaller veins draining localized septal territories may allow more controlled and targeted myocardial injury. In our limited experience, reduction in the LVOT gradient occurred immediately after ethanol infusion, similar to the hemodynamic response typically observed after conventional arterial SAA.^4^^,^^10^

Based on current knowledge, arterial SAA should remain the primary catheter-based septal reduction strategy when suitable septal perforator arteries are present. Venous SAA may represent an adjunctive or salvage technique in selected patients when arterial anatomy is unfavorable or when prior arterial septal ablation has failed.

## Conclusions

In patients with obstructive HCM who exhibit inadequate response to arterial SAA, particularly those with prior septal artery occlusion, venous SAA may represent a feasible salvage strategy in experienced centers. Further studies are needed to define optimal patient selection, safety, and long-term outcomes.

**Visual Summary:**
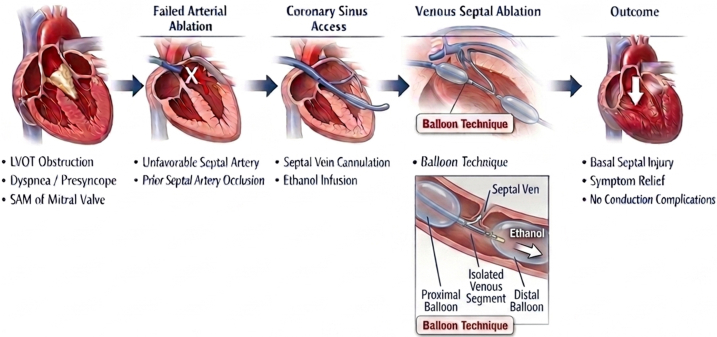
Venous Septal Alcohol Ablation as a Salvage Strategy in Obstructive Hypertrophic Cardiomyopathy When conventional arterial septal alcohol ablation is ineffective or not feasible because of unfavorable septal artery anatomy or prior septal artery occlusion, ethanol can be delivered through the coronary venous system to target the basal septum and reduce LVOT obstruction. LVOT = left ventricular outflow tract; SAM = systolic anterior motion.

## Funding Support and Author Disclosures

The authors have reported that they have no relationships relevant to the contents of this paper to disclose.

**Take-Home Messages**

• Venous SAA serves as a viable salvage strategy for septal reduction when conventional arterial access is anatomically precluded or ineffective because of a previous vessel occlusion.
• Controlled ethanol delivery via the coronary venous system, using either direct cannulation or balloon-isolated segments, can effectively achieve hemodynamic improvement and symptomatic relief in obstructive hypertrophic cardiomyopathy.
